# A new mechanism of POCD caused by sevoflurane in mice: cognitive impairment induced by cross-dysfunction of iron and glucose metabolism

**DOI:** 10.18632/aging.203544

**Published:** 2021-09-21

**Authors:** Xing Ge, Yong Zuo, Jinhong Xie, Xincheng Li, Yan Li, Anand Thirupathi, Peng Yu, Guofen Gao, Changhao Zhou, Yanzhong Chang, Zhenhua Shi

**Affiliations:** 1 Laboratory of Molecular Iron Metabolism, College of Life Science, Hebei Normal University, Shijiazhuang 050024, Hebei Province, China; 2 Faculty of Sports Science, Ningbo University, Ningbo 315211, Zhejiang Province, China; 3 The First Hospital of Hebei Medical University, Shijiazhuang 050000, Hebei Province, China

**Keywords:** sevoflurane, cognitive impairment, POCD, iron metabolism, glucose metabolism

## Abstract

Sevoflurane (Sev) is a commonly used anesthetic in hospitals that can cause neurotoxicity. Postoperative cognitive dysfunction (POCD) is a common clinical problem induced by some anesthetics. However, the exact mechanism of neurotoxicity induced by Sev is unclear. Here we studied a new mechanism of POCD induced by Sev. We treated 15-month-old mice with 2% Sev for 6 hours, and we had found that Sev causes POCD. Using isobaric tags for relative and absolute quantitation (iTRAQ), we found that the transporter and the metabolism of carbohydrates and inorganic ions were involved in the cognitive impairment induced by Sev. Using synchrotron radiation micro-X-ray fluorescence (μ-XRF), we showed that Sev caused the iron overload in the brain of 15-month-old mice. Subsequently, excessive iron led to oxidative stress and impaired mitochondrial function that further led to glucose metabolism disorder and reduced ATP production by regulating the expression of key enzyme genes or proteins including G6Pase, Pck1, and Cs. Meanwhile, Sev also inhibited the oxygen consumption rate and glucose absorption by downregulating the expression of glucose transporter 1 in cerebral vascular endothelial cells. The cross-dysfunction of iron and glucose metabolism caused the apoptosis in the cortex and hippocampus through Bcl2/Bax pathway. In conclusion, the data here showed a new mechanism that Sev caused apoptosis by cross-dysregulation of iron and glucose metabolism and induced energy stress in mice. Maintaining iron and glucose metabolism homeostasis may play an important role in cognitive impairment induced by Sev.

## INTRODUCTION

Every year, millions of patients require surgical procedures and anesthesia worldwide. Although anesthetics are considered safe to use, some of their side effects induced by anesthetics have attracted widespread attention in recent years. One of the most common types of side effects is cognitive impairment such as postoperative cognitive dysfunction (POCD). POCD is a major issue induced by anesthetics especially in aged patients, with the characteristics of anxiety and memory impairment [1]. It was reported that the incidence of POCD was 26% at 1 week and 10% at 3 months [2]. A growing body of evidence suggested that anesthesia was a high-risk factor for POCD whether in elderly patients [3–5] or in animal models [6, 7]. There is an ongoing public health concern about POCD as a result of anesthesia, and thus a hypothesis has been proposed that exposure to anesthetics, especially to general inhaled anesthetics, accelerates POCD neuropathology [4, 8].

Sevoflurane (Sev) is a commonly used inhalation anesthetic in hospitals that can induce POCD through several signaling pathways. In recent years, some reports suggested that Sev could induce neuronal apoptosis and the resulting neurotoxicity is attributed to cognitive impairment like POCD in the elderly animal models [1, 9, 10]. In addition, a few researches showed that reactive oxygen species (ROS) and neuroinflammation [11, 12–15] were closely related to the pathogenesis of POCD. Despite more than 20 years of intensive research and the fact that some signaling pathways were discovered, most details and exact mechanisms about POCD remain unclear.

Iron, as a cofactor of some electron transport complexes in the mitochondrial respiratory chain, plays an important role in energy production during the glycolysis and tricarboxylic acid (TCA) cycle. Glucose-6-phosphatase (G6Pase) could be inhibited by iron overload via heme catabolism by HO-1 that generates labile Fe, which represses gene G6Pase transcription in liver [16]. This indicated that iron overload influenced the glucose metabolism. Adenosine triphosphoric acid (ATP) produced by glucose catabolism plays a critical role in brain tissues. ATP production from glucose metabolism is controlled by some proteins or some key enzymes such as glucose transporter, pyruvate kinase M1(PKM1), phosphoenolpyruvate carboxykinase1(Pck1), glucose-6-phosphatase, citrate synthase (Cs), and cis-aconitase. Suzuki reported that Sev impaired insulin secretion and inhibited the glucose-induced increase in ATP levels in MIN6 cell line [17]. Coenzyme Q (COQ) is one of the important components of mitochondrial respiratory chain. Coenzyme Q10 decrease Sev-induced cognitive deficiency in mice [18], which implies that Sev possibly influenced the energy metabolism. However, the exact relationship among the cognitive deficiency pathology, abnormal iron metabolism, ATP production, and glucose metabolism induced by Sev in the brain is not fully understood. Here, using the isobaric tags for relative and absolute quantitation (iTRAQ) to investigate the differential protein expression and associated signaling pathways, we found that inorganic ions and glucose metabolism pathways are involved in Sev-induced cognitive impairment. Considering the role of iron in energy production, we tested the hypothesis that anesthetics could cause a cognitive impairment via dysfunction of iron metabolism, and further induced glucose metabolic disturbance. We found that Sev promoted the pathological feature of POCD in aged mice by causing iron and glucose metabolic dysfunction.

## RESULTS

### Sev can induce the occurrence of POCD symptoms in mice

**Figure 1:**
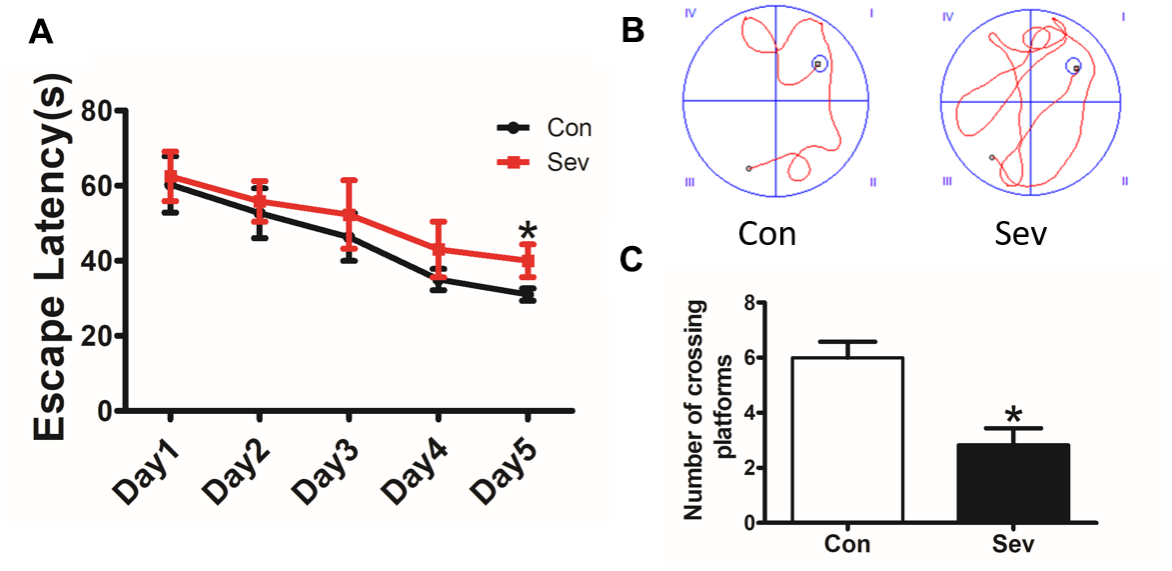
**Sev decreased the cognitive function of elder mice.** The mice were treated with Sev as described in the Materials and Methods section. The cognitive function was detected by the MWM test. The results were calculated as the escape latency (sec.) and platform crossing time ± SD (n = 15). (**A**, **C**). (**B**) Shows the typical movement locus. *P < 0.05 compared with the control group by one-way ANOVA.

One of the main symptoms of POCD is impaired memory. To test the effect of Sev on the memory of mice, we chose 15-month-old mice as test subjects. The results showed that the time taken to find the platform by Sev group mice was significantly longer than that by control group mice on the fifth day of the water maze test, indicating that Sev significantly caused a decline in memory function of 15-month-old mice as shown in Figure 1A, 1B. Meanwhile, the number of mice crossing the platform in Sev group mice decreased significantly on the sixth day compared with that of control group mice as shown in Figure 1C. The results suggested that Sev can induce the occurrence of POCD symptoms in aged mice.

### Analysis of eukaryotic orthologous groups (KOG) from differentially expressed proteins and Kyoto encyclopedia of genes and genomes (KEGG)

**Figure 2:**
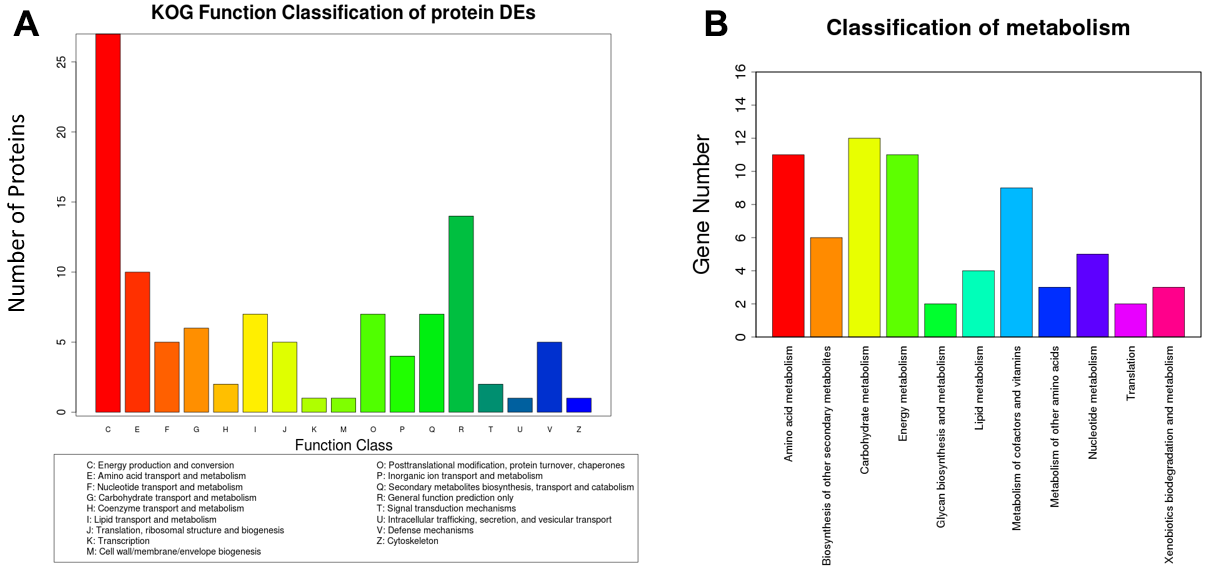
**Analysis of eukaryotic orthologous groups (KOG) and classification of metabolism.** The mice were treated with Sev as described in Materials and Methods. The data was from hippocampus tissues. (**A**, **B**) shows the KOG function classification of proteins and metabolism induced by Sev compared with control group, respectively.

In order to research the role of functional proteins and metabolic pathways associated with cognitive function in aged mice, we conducted BLAST comparisons between the sequences of differentially expressed proteins in hippocampus treated with Sev based on the information of iTRAQ and the KOG database from NCBI. We deemed the proteins that were upregulated or downregulated by ≥1.5 times, with a confidence coefficient >95% or unused Prot Score >1.3, as significantly changed. Based on a percent identity >30%, e-value <1e-10 and coverage >40% as the screening standard, we found 92 homologous proteins from the KOG database. These proteins are mainly involved in energy production and conversion, as well as transporter and metabolism of amino acids, carbohydrates, inorganic ions, and so on, as shown in Figure 2A. For KEGG analysis, InterProScan analysis was performed on the identified differential proteins from the data of iTRAQ. We identified a total of 52 pathways that were involved in 39 proteins. The main pathways included amino acid metabolism, biosynthesis of other secondary metabolisms, carbohydrate metabolism, and energy metabolism, etc., as shown in Figure 2B. These data suggested that Sev-induced cognitive impairment may be mediated by multiple targets.

### Sev induces the dysfunction of iron metabolism in brain of 15-month-old mice

**Figure 3:**
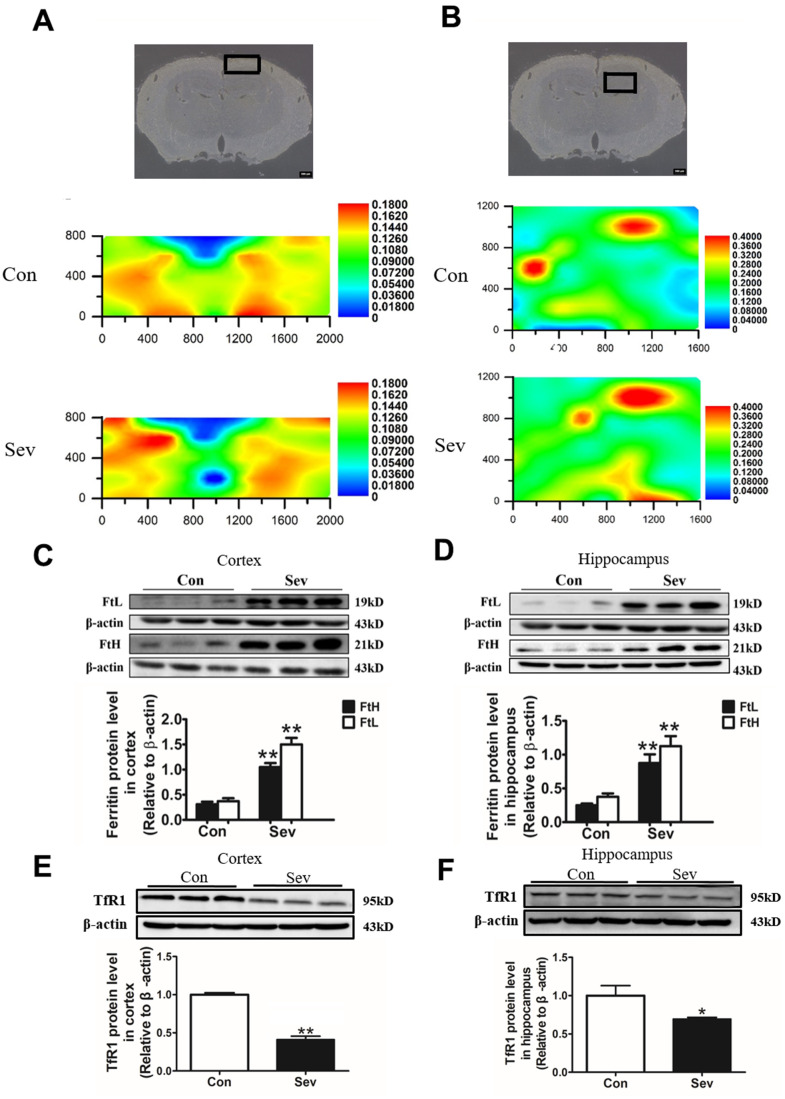
**Effect of Sev on the levels of brain iron and the expression of iron metabolism-related proteins in elder mice.** The mice were treated with Sev as described in Materials and Methods. (**A**, **B**) showed the iron levels of cortex and hippocampus of mice between control group and Sev group (The larger the red area, the greater the iron content). (**C**–**F**) showed the effect of Sev on the expression of FtL, FtH, and TfR1 in cortex and hippocampus. The western blot results were calculated as the ratios ± SD (n = 5) of the FtL, FtH, and TfR1 band intensities relative to that of actin. **P < 0.01 and *P < 0.05 compared with the control group by ANOVA, respectively.

Our previous study had reported that Sev could cause iron accumulation in brain in 15-month old mice [19]. However, we also found Sev-induced cognitive impairment in the offspring caused by iron deficiency during pregnancy in mice [20]. These results indicated that Sev had different effect on iron metabolism in different ages of mice. Based on the results of iTRAQ above and the effect of iron on the energy metabolism, here, we tested the iron content of cortex and hippocampus tissues exposure to Sev using μ-XRF. The results showed that Sev also caused iron overload in the cortex and hippocampus of 15-month-old mice as shown in Figure 3A, 3B. In order to verify the results of μ-XRF, we further tested the expression of ferritin and TfR1 in the cortex and hippocampus using western blot which are important proteins related to iron metabolism. We observed that Sev significantly increased the expression of L-ferritin and H-ferritin and decreased the expression of transferrin receptor 1 (TfR1) in both the cortex and hippocampus of 15-month-old mice. These findings indicated that Sev caused the dysfunction in iron metabolism in the neural tissues of 15-month-old mice, as shown in Figure 3C–3F.

### Sev impaired mitochondrial function and induced apoptosis through the ROS pathway in the brain

**Figure 4:**
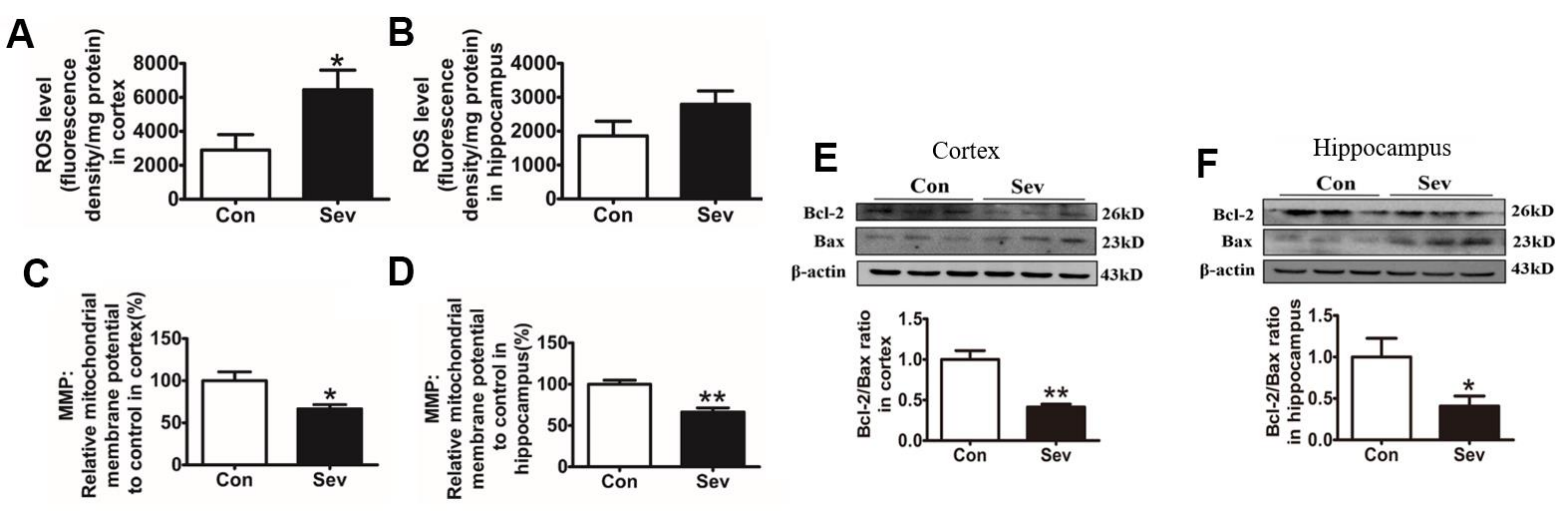
**Sev increased ROS production, reduced mitochondrial membrane potential, and promoted cell apoptosis.** The mice were treated with Sev as described in Materials and Methods. (**A**, **B**) Showed the ROS levels in cortex and hippocampus. (**C**, **D**) Showed the MMP levels in cortex and hippocampus. (**E**, **F**) Showed the expression of Bcl2, Bax and the ratio of Bcl2/Bax. Data are expressed as mean±SD, n=5 (**p<0.01vs.control group, *p<0.05 vs. control group).

The high level of ROS is the key factor in inducing cell apoptosis and the expression of anti-apoptotic and pro-apoptotic proteins reflecting the apoptotic state of cells. Mitochondrial membrane potential (MMP) is an important indicator of mitochondrial function. To investigate the effect of iron metabolism disorder on cell apoptosis and mitochondrial function in the hippocampus and cortex, we tested ROS production and the expression of apoptosis-related protein Bcl2 and Bax, and MMP levels. The results showed that Sev significantly increased the ROS production in cortex and decreased MMP in the cortex and hippocampus when compared to control group indicating that Sev impaired mitochondrial function through ROS pathway as shown in Figure 4A–4D. Meanwhile, our results showed that Sev also promoted the apoptosis through the Bcl2 and Bax pathway in brain as shown in Figure 4E, 4F.

### Sev decreased the content of ATP, inhibited the activity of Na^+^/K^+^ATPase, and reduced the OCR in cerebral microvascular endothelial cells

**Figure 5:**
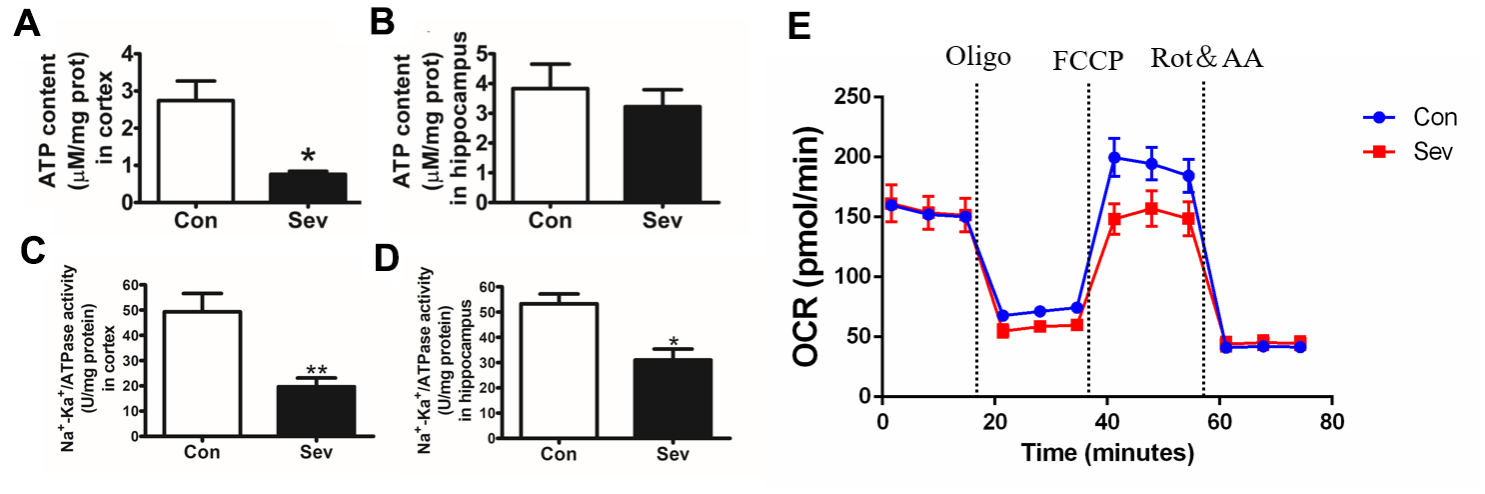
**Sev decreased the production of ATP, inhibited the activity of Na+/K+ATPase and changed OCR.** (**A**, **B**) showed the ATP production in cortex and hippocampus tested by ATP assay kit. (**C**, **D**) represented the Na+/K+ATPase activity in cortex and hippocampus, respectively. (**E**) The OCR was determined using Seahorse XFp8 as described in the Materials and Methods section. Data represented mean ± SD, n=5 (**p<0.01vs. control group, *p<0.05 vs. control group).

ATP generation and utilization is the core of energy metabolism in the brain. Na+/K+ATPase exerts an important role in maintaining the transmembrane gradient of Na+ and K+ ions inside and outside the cell and in the conduction of nerve impulses. Here, we investigated the effects of sevoflurane on ATP generation and ATP synthase activity. Our result showed that sevoflurane significantly reduced the amount of ATP in the cortex and the activity of Na+/K+ATPase in the cortex and hippocampus. Sevoflurane also reduced the content of ATP in the hippocampus, but there was no significant difference compared with the control group as shown in Figure 5A–5D. The microvascular endothelial cell controlled the absorption of glucose into brain tissue and its metabolic state affected the production and utilization of energy in the brain. Thus, we tested the OCR induced by Sev in microvascular endothelial cell bEnd.3. Our results showed that Sev significantly reduced the OCR indicating that Sev reduced the ability of endothelial cells to respond to energy requirements as shown in Figure 5E.

### Sev inhibited glucose metabolism by regulating the expression of some key enzymes

**Figure 6:**
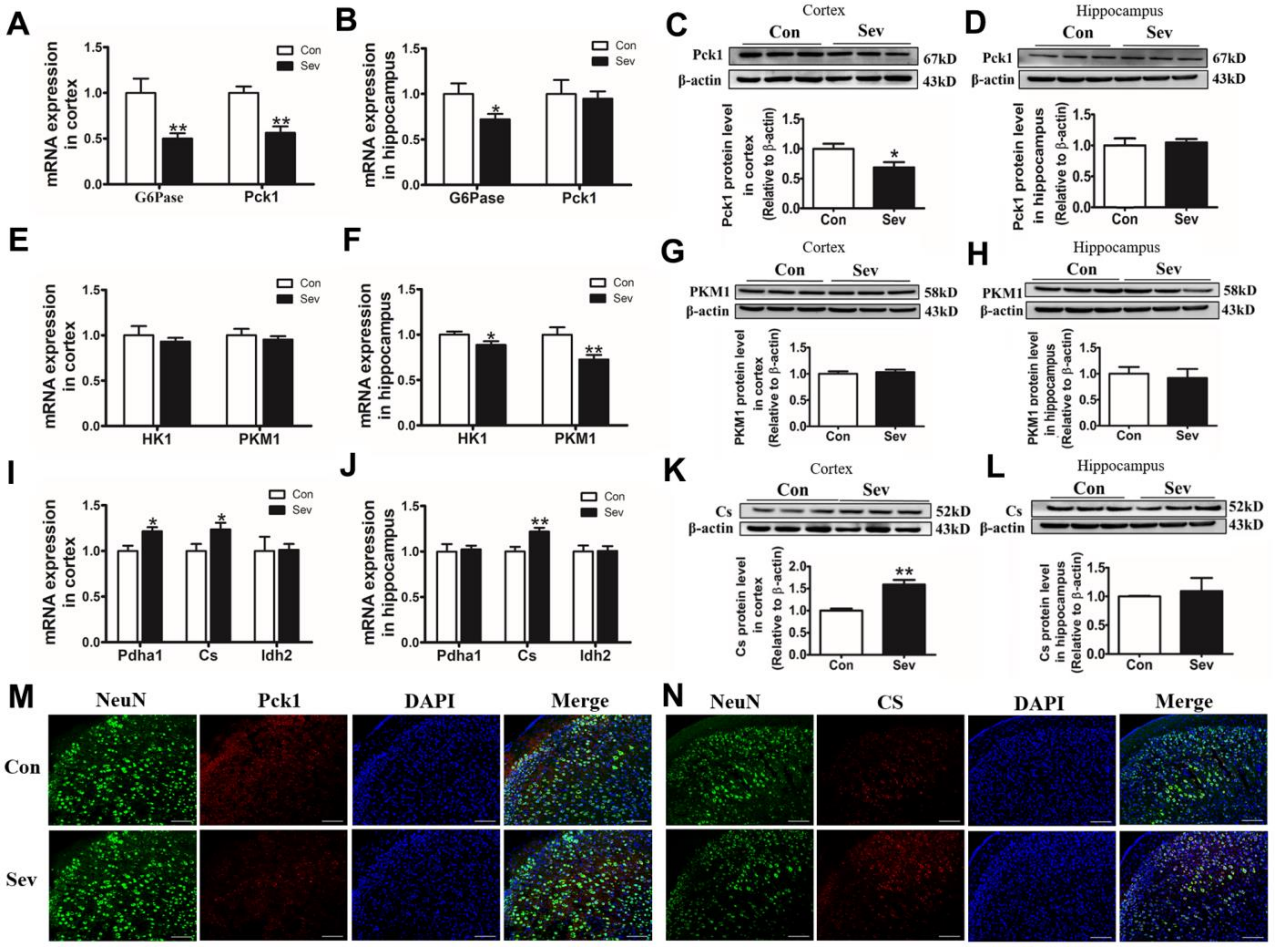
**Sev downregulated or increased the expression of key enzyme genes or proteins in glucose metabolism in the cortex or hippocampus.** The mRNA expression of G6Pase and Pck1 (**A**, **B**); HK1 and PKM1 (**E**, **F**); Pdha1, Idh2, and Cs (**I**, **J**) in cortex and hippocampus was measured by real-time PCR. The expression of Pck1 (**C**, **D**), PKM1 (**G**, **H**) and Cs (**K**, **L**) protein in cortex and hippocampus was tested by western blot. Data are expressed as mean ±SD, n=5 (**p<0.01vs. control group, *p<0.05 vs. control group). (**M**, **N**) Showed the expression of Pck1 and Cs in cortex by immunofluorescence, respectively.

Glucose metabolism involves the synthesis of glucose by gluconeogenesis and its decomposition by glycolysis and tricarboxylic acid cycle (TCA), both of which are involved in ATP generation and utilization. ATP content can affect glucose metabolism by regulating key enzymes in both processes. To examine whether Sev influenced ATP production by regulating the content of some key enzymes in the glycolytic pathways, we detected the expression of related key enzymes at the protein or gene level. We found that Sev significantly reduced the expression of G6Pase, Pck1, HK1, and PMK1in mRNA or protein levels in cortex or hippocampus compared with that of control group. However, changes in the expression of these genes or proteins were not consistent in the cortex and hippocampus as shown in Figure 6A–6H. The aerobic oxidation of glucose produces large amounts of ATP in the mitochondria through TCA pathway. Our results showed that Sev also affected the TCA cycle by regulating some key enzymes. Sev significantly upregulated the expression of Pdha1and Cs and it had no effect on the expression of Idh2 in cortex. However, Sev only increased the expression of Cs mRNA in hippocampus as shown in Figure 6I–6N These data indicated that Sev had a greater effect on the aerobic metabolism of sugar in cortical tissues than on the hippocampus.

### Sev inhibits the absorption of glucose by the Glut1 pathway

**Figure 7:**
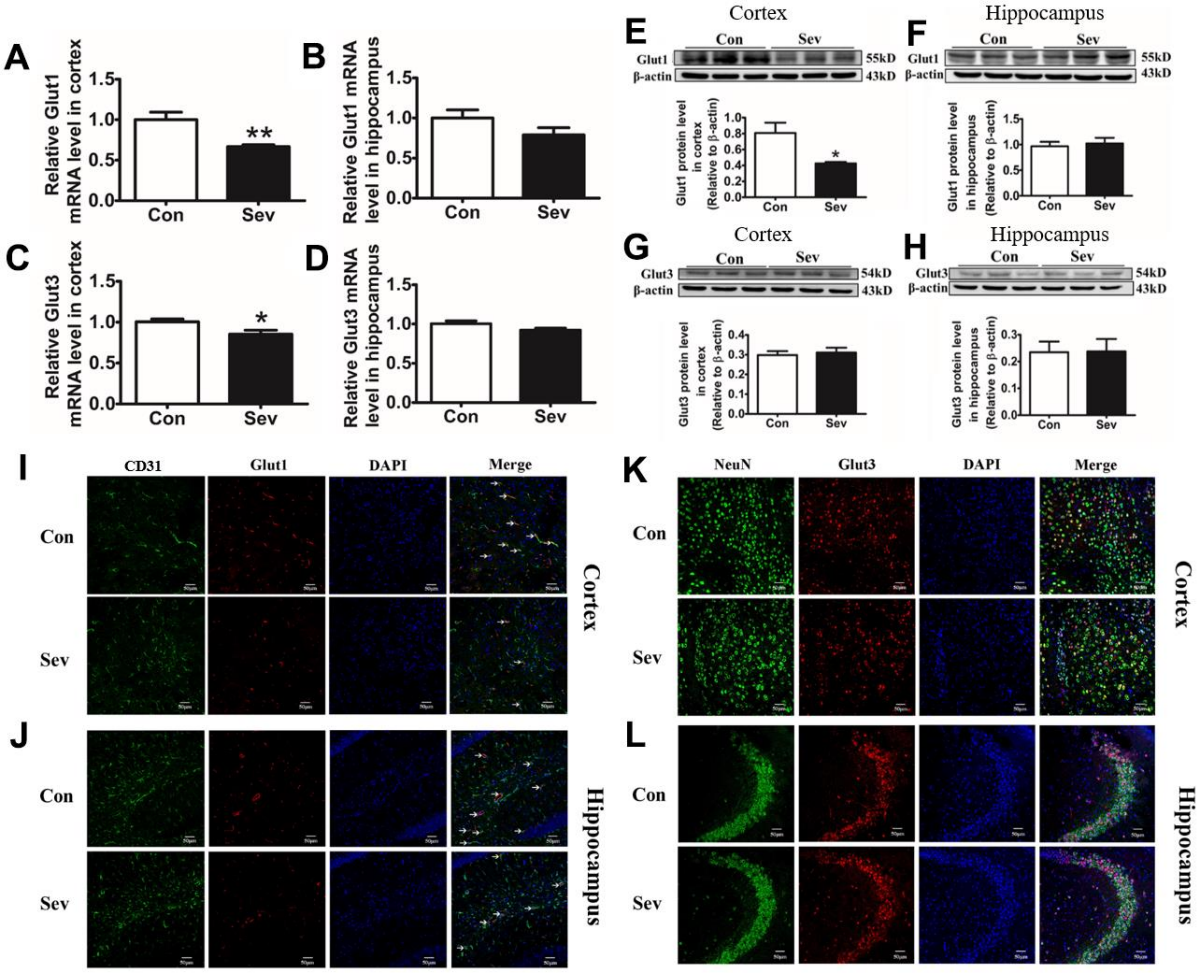
**Sev inhibited the absorption of glucose by downregulating the expression of Glut1 in vascular endothelial cells, rather than glucose receptor 3 in nerve cells.** Effect of Sev on the mRNA expression of Glut1 (**A**, **B**) and Glut3 (**C**, **D**) and the protein expression of Glut1 (**E**, **F**) and Glut3 (**G**, **H**) in cortex and hippocampus. (**I**–**L**) Showed the Glut1 and Glut3 expression in vascular endothelial cells and neurons of cortex and hippocampus by immunofluorescence, respectively. Data are expressed as mean±SD, n=5 (**p<0.01vs. control group, *p<0.05 vs. control group).

The absorption of glucose was a key step in glucose catabolism. To clarify whether Sev weakened the catabolism of glucose by inhibiting glucose absorption, we used qRT-PCR, western blot, and immunofluorescence to assay the expression of Glut1 and Glut3. We found that Sev significantly decreased the expression of Glut1and Glut3 of mRNA in cortex, but Sev had no effect on expression of these two genes in hippocampus. At the protein level, Sev significantly only downregulated Glut1 expression in cortex. However, Sev had no effect on expression of Glut3 in the cortex and Glut1 in hippocampus as shown in Figure 7A–7H. In order to further verify the results, we used immunofluorescence to test the expression of Glut1 in cerebral microvascular endothelial cells and Glut3 in neurons. The results showed that Sev indeed decreased the Glut1 and had no effect on Glut3 expression as shown in Figure 7I–7L. These data indicated that Sev mainly inhibits the absorption of glucose by the Glut1 pathway which was consistent with OCR.

### Sev inhibits the phosphorylation of AKT

**Figure 8:**
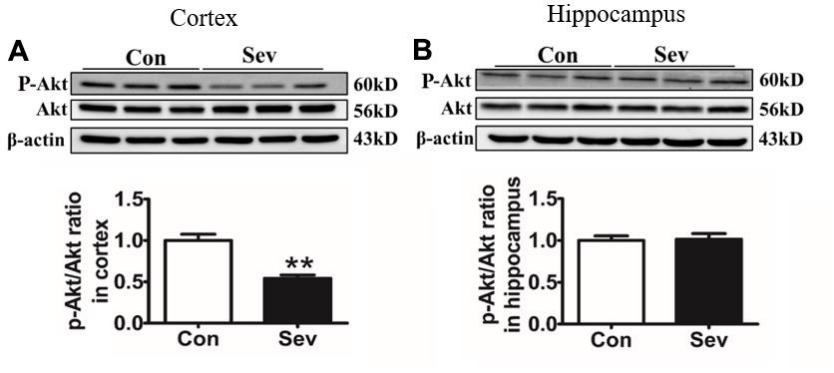
**Sev significantly inhibited the phosphorylation of Akt in the cortex rather than the hippocampus.** Effect of Sev on the expression of P-Akt in cortex (**A**) and hippocampus (**B**). Data are expressed as mean±SD, n=5 (**p<0.01vs. control group).

The AKT signaling pathway exerts an important regulatory role in glycometabolism [21]. Moreover, iron levels in cells are closely related to AKT phosphorylation [22]. In order to test whether AKT pathway was involved in regulating glucose metabolism under conditions of iron metabolism disorder induced by Sev, we measured the phosphorylation levels of AKT. We found that Sev significantly inhibited the phosphorylation of AKT in cortex compared with that of control group. However, Sev had no effect of AKT phosphorylation in hippocampus as shown in Figure 8, indicating that Sev inhibits AKT phosphorylation by inducing high iron levels in the cortex, thus reducing glucose metabolism.

## DISCUSSION

Anesthetics are necessary drugs in clinical operation. However, some side effects caused by anesthetics have attracted more and more attention for decades. A growing body of research shows exposure to anesthetics leads to neurotoxicity and apoptosis of nerve cells, which in turn induces cognitive impairment such as POCD [2, 4]. Sev is one of the most commonly used inhalation anesthetics and has been implicated in both POCD and neurotoxicity.

Although many mechanisms, including apoptosis, neuroinflammation, oxidative stress, and autophagy, have been found to be involved in Sev induced neurotoxicity, the exact molecular mechanisms remain unknown. Here, using iTRAQ, we found that Sev induced cognitive impairment like POCD symptoms which involved several metabolic pathways including the generation and conversion of energy, amino acid transport, glucose and inorganic ion metabolism, and so on, in 15-month-old mice as shown in Figures 1, 2. We recently completed the work demonstrating that sevoflurane causes brain iron deficiency, inhibits myelin development, and induces cognitive impairment in the offspring of pregnant mice [20]. However, our recent and others study have shown that Sev can cause cognitive impairment by inducing the iron overload in brain in 12-month-old mice [19, 23]. This suggests that Sev has different effects on iron metabolism in mice of different ages. Considering that Fe-S protein in the respiratory chain of mitochondrial plays a key role in electron transport and energy production, we hypothesized that Sev might affect iron metabolism disorders and function of respiratory chain in mitochondrial Thus, in order to identify the iron metabolism of cortex and hippocampus, we used μ-XRF to detect the levels of cortex and hippocampus. Our results showed that Sev caused the iron levels raised significantly in both cortex and hippocampus mice. Ferritin and TfR1 are two important proteins that reflect iron metabolic homeostasis. When cellular iron levels increased, ferritin expression is significantly upregulated and the expression of TfR1 would decrease by IRE/IRP regulative system. Our results of western blot further proved that Sev significantly increased the expression of FtL and downregulated the expression of TfR1, indicating that Sev induced the dysfunction of iron metabolism in cortex and hippocampus as shown in Figure 3. Wu [23] recently also confirmed that iron overload was involved in the pathogenesis of sevoflurane-induced neurotoxicity and cognitive deficits indicating that Sev-induced neurotoxicity and iron metabolism disorders are closely related. Too much iron initiated the oxidative stress pathway and produced amount of ROS, especially in cortex through the Fenton reaction, and further damaged the function of mitochondrial via decrease the MMP, inducing cell apoptosis via Bcl2/Bax pathway as shown in Figure 4.

Iron homeostasis is closely related to glucose metabolism [24, 25]. Clinical studies suggested that deregulation of iron metabolism in iron overload disorders is associated with other metabolic dysfunction [26].

Glucose is the main source of energy for the brain. If glucose is lacking, neurotransmitters are not synthesized and communication between neurons breaks down. To detect the effect of Sev-induced iron overload on energy production in brain, we measured the ATP content and Na+/K+ ATPase activity in the cortex and hippocampus. We found that Sev significantly decreased the ATP content and Na+/K+ ATPase activity especially in cortex. ATP levels and Na+/K+ ATPase activity provided the driving force for the coordinated transport of glucose. Decrease in ATP levels and Na+/K+ ATPase activity indicated that Sev inhibited the intake of glucose in the brain. Energy stress depletes ATP and induces cell death [27]. Decrease of ATP content induced by Sev could be the important reason of nerve cell apoptosis.

Cerebral vascular endothelial cells play a key role in the entry of glucose into brain tissue. OCR is an important indicator of mitochondrial respiration capacity. According to our OCR data, Sev attenuated the reduction of ATP production-linked OCR and maximal respiration in bEnd.3 cells which is a kind of vascular endothelial cell from mouse. This is consistent with decrease in ATP content of brain. Glucose metabolism regulated the energy demand of the brain through the expression or allosteric effect of some key enzymes. These key enzymes included G6Pase, Pck1 which was responsible for gluconeogenesis, and HK1, PKM1, Pdha1, Cs, and Idh2 which was responsible for glucose catabolism through TCA cycle. Our data showed Sev inhibited the expression of G6Pase and Pck1 levels, and increased the expression of Pdha1, Cs, and Idh2 in mRNA or protein in cortex or hippocampus, respectively, indicating that Sev indeed caused the shortage of ATP in brain.

The absorption of glucose in the brain depends on the glucose transporters (Glut). Glut1 and Glut3 were mainly expressed in cerebral microvascular endothelial cells of blood brain barrier (BBB) and neurons, respectively [28]. Glut1 controlled the passage way for glucose into the brain and regulated the availability of glucose to the neurons. Tianyi Jiang et al. reported that the expression of Glut1 in microvascular endothelial cells decreased at old age [29]. In order to identify whether the decrease in ATP production in brain is caused by inhibiting glucose absorption in microvascular endothelial cells or neurons, we tested the expression of Gltu1 and Glut3 using qPCR and western blot. Our data showed that Sev significantly downregulated the expression of Glut1 in cerebral microvascular endothelial cells, but had no effect on the expression of Glut3. These effects are supported by the observation from immunofluorescence assay as shown in Figure 8I–8K, indicating that Sev mainly affected the glucose metabolism in elder mice brain through Glut1 pathway.

The AKT pathway exerts an important role in the regulation of glucose metabolism. Iron overload suppressed the AKT phosphorylation [30] which could cause neuronal metabolic defects [21]. On the contrary, iron deficiency promoted the AKT phosphorylation [22]. Here we demonstrated for the first time to our knowledge that overload of iron-induced by Sev inhibited the phosphorylation of AKT, which was one of the important causes of cerebral glucose metabolism disorder in mice.

**Figure 9:**
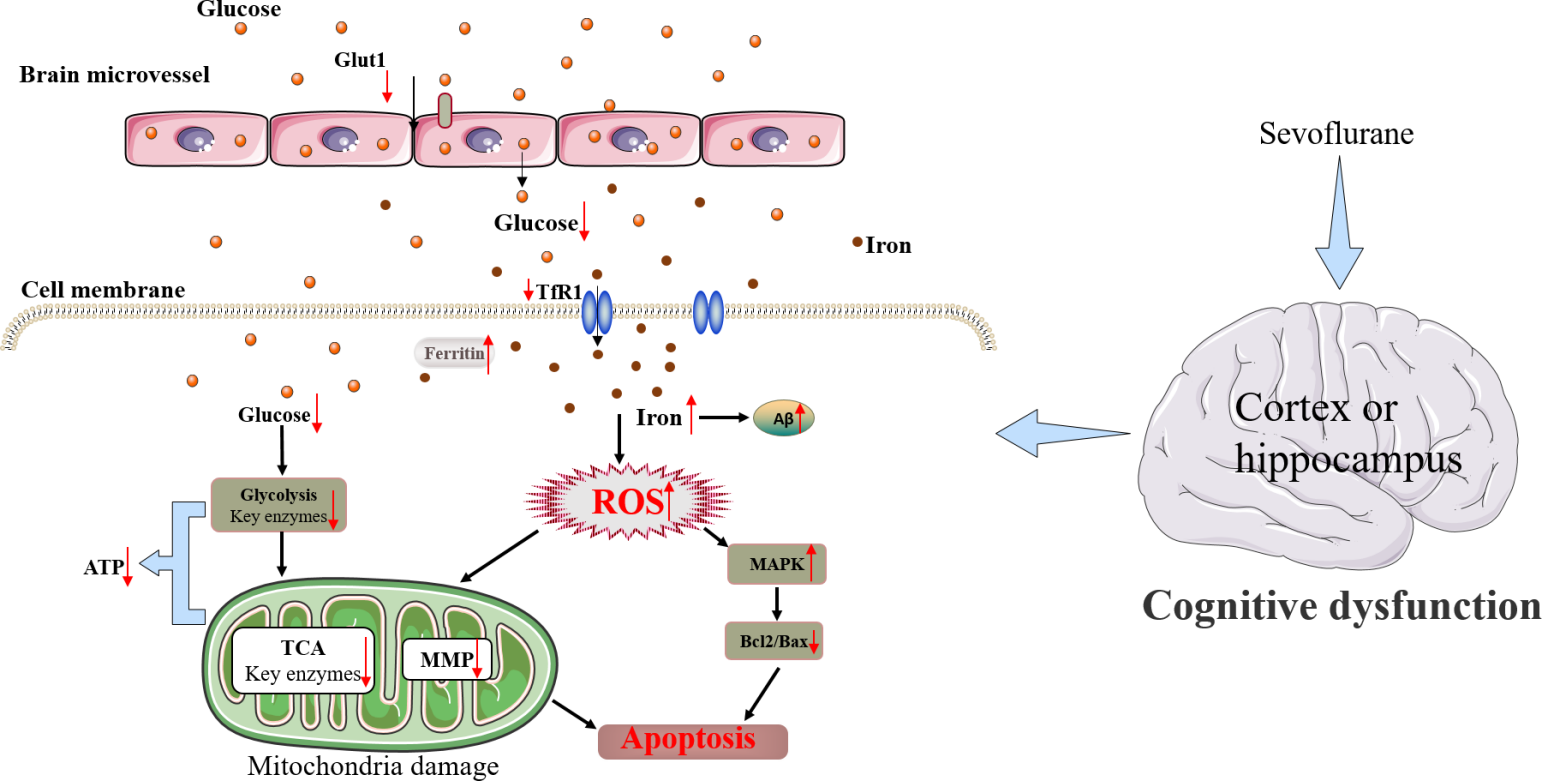
Schematic diagram of the neurotoxic effect of Sev in mice.

In conclusion, Sev may induce cognitive impairment in mice by causing iron overload which further promoted oxidative stress damage and inhibited uptake of glucose through cerebral microvascular endothelial cells, resulting in a shortage of energy in the brain. Although the mouse model has limitations in mimicking the human brain completely. Still, these results have important clinical implications. This study showed a new mechanism that cross-dysregulation of iron and glucose metabolism played an important role in cognitive impairment induced by Sev in mice. Based on our results here and combined with our previous research work [19, 31], we summarized the possible mechanism of nerve injury induced by sevoflurane as shown in Figure 9.

## MATERIALS AND METHODS

### Anesthesia treatment of mice

All animal experiments were performed in accordance with ethical standards and the procedures were approved by the Hebei normal university ethics committee. 15-month-old healthy C57BL/6 male mice were obtained from the Center of Experimental Animals, Hebei Medical University (Shijiazhuang, China). All the mice were fed in stainless steel rust-free cages at 22–24° C and a 12-h light/dark cycle and were provided free access to the food and distilled water.

A total of sixty 15-month-old C57BL/6 male mice were assigned into two groups: control group and Sev treatment group. Anesthesia treatment of mice was according to the previous report [19].

### Morris water maze (MWM) test

We used MWM to test the capacity of the mouse to learn and memory according to the previous report [19]. Briefly, for mice in control group, each mouse was given swimming training one time a day for 6 days. For Sev group mice, the mice were first anesthetized and then they were given the MWM test. Then, these mice were given swimming training one time a day for 6 days. On the sixth day, the platform was removed to test the times of crossing platform. For each mouse, the escape latency was recorded as 120 s.

### Assay of isobaric tags for relative and absolute quantitation (iTRAQs) labeling

Protein lysates of the hippocampus were obtained using ice-cold SDS buffer (0.1 mM Tris-HCl, pH 8.8, 2% (w/v) SDS, 1 mM EGTA, 10 mM EDTA). The protein concentrations were evaluated using a BCA Kit, and the samples were dried in a vacuum concentrator. The proteins were diluted to the proper concentration by ultrapure water. For iTRAQ labeling of peptides, the samples were mixed in the same tube and loaded on a C18 column (5 μm, 4.6 × 250 mm), followed by a mobile phase elution with buffer A (20 mM ammonium formate, pH10) and buffer B (20 mM ammonium formate 80% ACN or 100% ACN). The flow rate was 0.8 mL/min, and the sample was injected after the initial 20 min equilibration phase. Within 5 min of the injection, the eluate was collected throughout the run. The samples were dried in a vacuum concentrator for the analysis of mass spectrum, eukaryotic orthologous groups (KOG), and classification of metabolism.

### Determination of iron content in the cortex and hippocampus

The determination of iron content in cortex and hippocampus was done using synchrotron imaging according to our previous report [20]. Briefly, the coronal sections of frozen cortex and hippocampus were cut into 50-μm-thick sections and installed on a 3-mm-thick polycarbonate film. The sections were analyzed by synchrotron radiation micro-X-ray fluorescence (μ-XRF) which was on a 4W1B end station at the Beijing Synchrotron Radiation Facility, a 4W1B end station at the Beijing Synchrotron Radiation Facility, operating at 2.5GeV with a current from 150 mA to 250 mA. Finally, a Si (Li) solid-state detector was used to detect the X-ray fluorescence. Data processing was done using the PYMCA package.

### Assay of ROS

ROS levels were measured by Invitrogen ROS Assay Kit (Shanghai, China) according to protocols provided by the company. Briefly, hippocampus and cortex tissues were homogenized on ice using 1% Triton-100 in phosphate-buffer (pH7.4) with protease inhibitors (1μg /ml pepstatin A, 1μg /ml leupeptin, and1μg /ml aprotinin). Lysates were centrifuged at 10 300g for 5 min and then the supernatant was collected.

The total protein content was determined by BCA kit. 50 μl sample (triplicate) and 50 μl catalyst solution were added to a black 96-well plate, and then the mixture was incubated for 5 min at room temperature. Then 100 μl dichlorodihydrofluorescein (DCFH) solution was added to the samples, and the mixture was sequentially incubated for 20 min at room temperature. Finally, the fluorescence intensity was measured on a microplate reader (Synergy H4, Bio-Tek, Winooski, VT, USA) at excitation 480 nm and emission 530 nm.

### Mitochondrial membrane potential (MMP) detection

MMP was detected using tissue mitochondria isolation kit (C3606, Beyotime Company, China) and MMP assay kit with JC-1 (C2006, Beyotime Company, China), according to the previous report. Briefly, the mitochondria of cortex and hippocampus firstly were isolated according to the protocol provided by the company. Secondly, 0.9 mL JC-1 staining solution was added with 0.1 mL purified mitochondria with a total protein content of 10–100 μg. The fluorescence intensity was assayed using a microplate reader (Synergy H4, Bio-Tek, Winooski, VT, USA) at excitation 485 nm and emission 590 nm.

### Measurement of ATP contents

ATP content of tissue was tested by using ATP assay kit (S0026, Beyotime, China). Briefly, samples from hippocampus or cortex tissues were lysed by ATP releasing reagent. Then the extracts were mixed with ATP detection solution which contained luciferase, the bioluminescence was detected by Synergy HT luminescence plate reader. ATP content was evaluated according to the standard curve. Results were normalized to tissue protein concentration, which was determined by an enhanced BCA Protein Assay kit (Beyotime, China).

### Measurement of Na^+^/K^+^-ATPase activity

The cortex and hippocampus tissues were homogenized with 1% saline. After centrifugation at 4,000 g for 10 min, the activity of Na^+^ /K^+^-ATPase in the supernatant was evaluated spectrophotometrically with the Na+/K+-ATPase activity assay kit (A070-2, Nanjing Jiancheng Biochemistry Co, Nanjing, China). The protein concentration was detected by the BCA Protein Assay kit (Beyotime, China). The activity of Na^+^ /K^+^-ATPase was expressed as μ mol per milligram protein.

### Cell culture and oxygen consumption rate (OCR) test of cerebral vascular endothelial cells

Cerebral vascular endothelial bEnd.3 cells were cultured in Dulbecco’s modified Eagle’s Medium (DMEM; Gibco, Carlsbad, CA, USA) supplemented with 10% fetal bovine serum (Gibco) and 1% penicillin-streptomycin (Sigma-Aldrich, St. Louis, MO, USA), in a humidified atmosphere at 37° C under 5% CO2.

The OCR of bEnd.3 cells was detected by sequentially adding specific inhibitors of respiratory chain from the Seahorse XF Cell Mito Stress Test Kit (103010-100, Seahorse Bioscience, North Billerica, MA, USA) using the Seahorse XFp8 Extracellular Flux Analyzer, according to the manufacturer’s protocol. Briefly, the cells of Sev group were treated with 2% Sev for 6 h in an anesthetic chamber in a carbon dioxide incubator with 5% CO 2 at 37° C. Then the cells were incubated at a density of 2×10^4^ cells/well in XFp8 microplates. Before the detection, the cells were washed three times with assay medium (102353-100; Seahorse Bioscience) and then incubated in a CO_2_-free incubator at 37° C for 1 h. Finally, OCR was assayed by an XFp8 analyzer. Three inhibitors of respiratory chain, oligomycin (1.0 μM), carbonylcyanide-4-(trifluoro-methoxy) phenylhydrazone (FCCP; 1.0 μM), and a mixture of rotenone (Rot) and antimycin A (AA) (0.5 μM), were sequentially injected in the OCR measurement to obtain the values for the basal mitochondrial respiration, ATP-linked oxygen consumption rate, maximal respiration, and spare respiratory capacity.

### Real-time PCR

Total RNA from the hippocampus and cortex was extracted using Trizol (Invitrogen, Beijing, China). Real-time PCR was used to detect the expression of related genes.

One-step SuperScript kit (Life Technologies Beijing, China) for real time PCR was purchased from Life Technologies company (Beijing, China).

The quantity of G6Pase, Pck1, HK1, Pdha1, Cs, Idh2, Glut1, and Glut3 gene mRNA expression was detected with the PRISM ABI 7900HT Sequence Detection System (Applied Biosystems, Waltham, MA, USA). The primers used for genes above were as follows:

**Table:** 

**Name**	**Sense sequence**	**Anti-sense sequence**
G6Pase	5'-TGGAACCAGATGGGAAAGAG-3'	5'-AGGAAGGATGGAGGAAGGAA-3'
Pck1	5’-AACTGTTGGCTGGCTCTC-3’	5’-GAACCTGGCGTTGAATGC-3’
HK1	5’-GAACCTGGCGTTGAATGC-3’	5’-CCCACGGGTAATTTCTTGTCC-3’
PKM1	5’-GTGGCTCGGCTGAATTTCTCT-3’	5’-CACCGCAACAGGACGGTAG-3’
Pdha1	5’-TGGCAGCACTGTGGAAATTA-3’	5’-CGCACAAGATATCCATTCCA-3’
Cs	5’-GGAAGGCTAAGAACCCTTGG-3’	5’-TCATCTCCGTCATGCCATAGT-3’
Idh2	5’-TCATCTCCGTCATGCCATAGT-3’	5’-TCATCTCCGTCATGCCATAGT-3’
Glut1	5’-CAGTTCGGCTATAACACTGGTG-3’	5’-GCCCCCGACAGAGAAGATG-3’
Glut3	5’-TCATCAATGCACCTGAGACAATC-3’	5’-GTCCCTCACTTGGTAGGTCTT-3’

All PCRs were carried out in triplicate.

### Immunofluorescence assay

For immunofluorescence assay, the mice were anesthetized using 0.4% pentobarbital sodium (1 mL/100 g) solution. After that, the mice were perfused with 0.9% saline and then treated with 4% paraformaldehyde. The brains were carefully dissected and then were immersed in 30% sucrose solution for 2 days. The brain was sliced into 15-μm-thick slices, and then the slices were washed with 0.01 M PBS three times for 5 min each time. The slices were then incubated with goat serum at 37° C for 60 min. Mouse monoclonal anti-NeuN antibody (1:200; Cat. No. ab104224, Abcam, Waltham, MA, USA), anti-GLUT3 antibody (1:400; Cat. No. 20403-1-AP ProteinTech, Rosemont, IL, USA), anti- GLUT1 (1:400; No. ab115730 Abcam, Waltham, MA, USA), and anti-CD31 (1:400; Cat. No.ab24950, Abcam, Waltham, MA, USA) were used as primary antibodies. The slices were incubated overnight at 4° C. Next, the slices were incubated with FITC-conjugated and rhodamine-conjugated secondary antibodies. Images were observed using a ZEISS LSM710.

### Western blotting

The western blotting method was performed as previously described by Shi [32] et al. Briefly, the cortex and hippocampus tissues were homogenized and lysed with RIPA lysis buffer. The lysates were centrifuged at 12,000 × g at 4° C for 10 min. The supernatant was collected. Protein content of supernatant was evaluated by using the BCA kit (Pierce), and 30–50μg protein (each group) was separated by SDS–PAGE. Then proteins were transferred to nitrocellulose membranes. Blots were blocked with blocking buffer containing 5% fat-free milk, 0.1% Tween 20 in 0.1M TBS and incubated with primary antibody overnight at 4° C. Then the membrane was incubated with the secondary antibody for 1 h at room temperature and finally reacted with the chemiluminescent substrate (Pierce Biotechnology, Rockford, IL, USA). The proteins were detected using the enhanced chemiluminescence (ECL) and quantified by transmittance densitometry with Multi Gauge ver. 3.1 software (FUJI FILM Corporation, Tokyo, Japan).

### Statistical analysis

All experiments were repeated at least three times. One-way ANOVA was performed to evaluate overall significance, followed by post hoc Tukey tests corrected for multiple comparisons. Data are presented as the mean ± standard deviation (SD). A probability level of 95% (P < 0.05) was considered significant.
